# Functional differences in cerebral activation between slow wave-coupled and uncoupled sleep spindles

**DOI:** 10.3389/fnins.2022.1090045

**Published:** 2023-01-18

**Authors:** Daniel Baena, Zhuo Fang, Aaron Gibbings, Dylan Smith, Laura B. Ray, Julien Doyon, Adrian M. Owen, Stuart M. Fogel

**Affiliations:** ^1^Sleep Unit, University of Ottawa Institute of Mental Health Research at The Royal, Ottawa, ON, Canada; ^2^School of Psychology, University of Ottawa, Ottawa, ON, Canada; ^3^McConnell Brain Imaging Centre, McGill University, Montreal, QC, Canada; ^4^The Brain and Mind Institute, Western University, London, ON, Canada; ^5^Department of Physiology and Pharmacology, Western University, London, ON, Canada; ^6^University of Ottawa Brain and Mind Research Institute, Ottawa, ON, Canada

**Keywords:** EEG-fMRI, sleep, sleep spindles, NREM (non REM) sleep, brain activation

## Abstract

Spindles are often temporally coupled to slow waves (SW). These SW-spindle complexes have been implicated in memory consolidation that involves transfer of information from the hippocampus to the neocortex. However, spindles and SW, which are characteristic of NREM sleep, can occur as part of this complex, or in isolation. It is not clear whether dissociable parts of the brain are recruited when coupled to SW vs. when spindles or SW occur in isolation. Here, we tested differences in cerebral activation time-locked to uncoupled spindles, uncoupled SW and coupled SW-spindle complexes using simultaneous EEG-fMRI. Consistent with the “*active system model*,” we hypothesized that brain activations time-locked to coupled SW-spindles would preferentially occur in brain areas known to be critical for sleep-dependent memory consolidation. Our results show that coupled spindles and uncoupled spindles recruit distinct parts of the brain. Specifically, we found that hippocampal activation during sleep is not uniquely related to spindles. Rather, this process is primarily driven by SWs and SW-spindle coupling. In addition, we show that SW-spindle coupling is critical in the activation of the putamen. Importantly, SW-spindle coupling specifically recruited frontal areas in comparison to uncoupled spindles, which may be critical for the hippocampal-neocortical dialogue that preferentially occurs during sleep.

## Introduction

There is a large body of evidence indicating that the optimal transformation of newly acquired, labile memory traces into lasting and integrated memories is facilitated by sleep (Diekelmann and Born, 2010; Lewis and Durrant, 2011; Dudai et al., 2015; Witkowski et al., 2020). The process of memory consolidation involves transfer of information from the hippocampus to the neocortex (Wang and Morris, 2009; Eichenbaum, 2017; Takehara-Nishiuchi, 2020), and during sleep, this process is facilitated by hippocampal ripples. Ripples are involved in the repeated reactivation and replaying of newly-formed hippocampal-dependent memory traces (Buzsáki, 2015; Sadowski et al., 2016). Ripples, however, do not occur in isolation. Rather, they are part of slow wave–spindle–ripple complexes, whereby ripples are nested in the excitatory troughs of spindles, and spindles are nested preferentially, but not exclusively (Yordanova et al., 2017), in the excitatory troughs (“up-states”) of slow waves (Helfrich et al., 2019). The “*active system model”* of memory consolidation suggests that these three oscillations form a temporal hierarchy, which serves as an endogenous timing mechanism that supports the hippocampal-neocortical dialogue. This dialogue is required for the consolidation of new memories (Latchoumane et al., 2017; Klinzing et al., 2019). While it is not possible to record hippocampal ripples in humans without employing invasive techniques, slow wave-spindle (SW-SP) coupling can be measured from scalp recordings and is considered to be an index of memory reactivation during sleep (Schreiner et al., 2021).

Advances in simultaneous EEG-fMRI recording have enabled the measurement of regional brain activity and functional connectivity, time-locked precisely to spontaneous events during sleep, such as slow waves (Kaufmann et al., 2006; Dang-Vu et al., 2008; Betta et al., 2021) and spindles (Laufs et al., 2007; Schabus et al., 2007; Tyvaert et al., 2008; Andrade et al., 2011; Caporro et al., 2012; Fang et al., 2019, 2020), hence providing insights into systems-level changes in brain activity that reflect memory consolidation during sleep. These studies have shown that spindles do not only recruit areas necessary for spindle generation (e.g., thalamus), but interestingly, that they spontaneously recruit and reactivate brain areas known to support memory functions (e.g., putamen, hippocampus). At the cellular level, spindles are associated with large influxes of Ca^2+^, which facilitates neocortical long-term potentiation *via* synaptic plasticity (Sejnowski and Destexhe, 2000; Timofeev et al., 2002; Nevian and Sakmann, 2006). This effect is enhanced when spindles occur during SW up states (Niethard et al., 2018). Thus, the spaced and repeated action of SW-SP complexes appear to be an ideal mechanism to facilitate hippocampal-neocortical systems-level consolidation.

However, it is not clear whether these spontaneous activations occur during all spindle events, or specifically during coupled SW-SP complexes. The present study thus aimed to address this unanswered question. We also investigated whether coupled spindles and uncoupled (i.e., isolated) spindles are dissociable from one another in terms of their functional neuroanatomical substrates. Here, we expected to observe dissociable patterns of recruited brain activity in areas time-locked to SW-SP coupled events, as compared to uncoupled spindle events, or compared to uncoupled slow wave events. We hypothesize that brain activations time-locked to coupled SW-spindles would preferentially occur in brain areas known to be critical for the hippocampal-neocortical dialogue and the striatum, required for the consolidation of new memories.

## Materials and methods

### Participants

All participants were 20–35 years of age. Participants were initially screened for irregular sleep schedules (bedtime outside the hours of ∼22:00–24:00 h, wake time outside the hours of 07:00–09:00 h), left-handedness, shift work, and the use of medications known to affect sleep. Participants were also excluded from the study if they considered themselves a smoker, consumed >1–2 caffeinated beverages/day, consumed >7 alcoholic beverages/week, or had a history of chronic pain, seizures or head injury. Participants were required to refrain from recreational drug use and limit caffeine (to no more than 1 beverage in the A.M.) and alcohol intake at least 3 days prior to, and throughout participation in the study. Actigraphy and sleep logs were used to confirm the participants’ sleep and activity cycles throughout the study. To rule out participants with signs of depression or anxiety and ensure normal sleep-wake patterns, participants also completed the Beck Depression (Beck et al., 1988b) and Anxiety Inventories (Beck et al., 1988a) as well as the Sleep Disorders Questionnaire (Douglass et al., 1994).

A total of 35 participants met study inclusion criteria. A minimum of 30 coupled and 30 uncoupled spindle events were necessary for analysis purposes. An additional fourteen participants did not meet this criterion. In total, *N* = 21 participants (mean age 24, 13 females) were included in the final data analyses.

### Ethics statement

All study procedures and methods adhered to the Declaration of Helsinki and were approved by the Western University Health Science research ethics board. All participants were given a letter with details of the study, provided informed consent, and were financially compensated for their participation.

### Procedures

**FIGURE 1:**
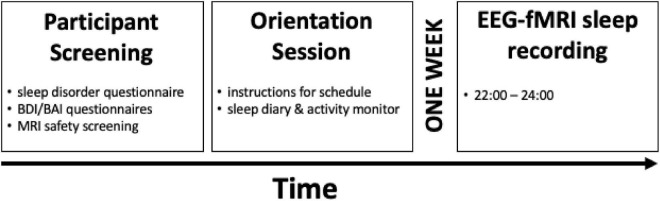
Study design. Participants underwent an initial screening to rule out any signs of sleep disorders, unusual sleep habits, or other health-related criteria and MRI compatibility. Eligible participants then visited the sleep laboratory for the orientation session at least 1 week before the EEG-fMRI sleep recording night, in which participants were given detailed instructions about the study procedure, the sleep diary, and an activity monitor. Finally, participants completed the EEG-fMRI sleep recording session beginning at 9 P.M, with lights out for the sleep session at 10 P.M. The sleep session ended at 12 A.M. Reproduced with permission from cerebral cortex.

All participants who met the study inclusion criteria underwent an orientation session where they were given the study instructions, a sleep diary and an activity monitor to verify their sleep–wake cycle (Figure 1). A minimum of 1 week following the orientation session, participants completed the EEG-fMRI sleep recording night. Participants arrived at the sleep laboratory on the recording night at approximately 8 P.M. The scanning procedures began at 9 P.M., at which point, the EEG equipment was installed and configured. Following this, localizer scans, a T1 structural scan, and an 8-min eyes-closed awake resting-state scan were completed. EEG was acquired during the initial scanning procedures to confirm that participants remained awake. These procedures took up to one hour to complete. The sleep session (“lights out”) began at about 10 P.M., within the range of the participants’ habitual bedtime. After the sleep session, participants slept the rest of the night in the nearby sleep laboratory.

### Polysomnographic acquisition and analysis

#### Polysomnographic recording parameters

Polysomnographic (PSG) recordings were obtained using a 64-channel magnetic resonance (MR)-compatible EEG cap, which included one electrocardiogram (ECG) lead (Braincap MR, Easycap) and 64-channels of EEG recorded *via* two MR-compatible 32-channel amplifiers (Brainamp MR Plus, Brain Products GmbH). EEG recordings were referenced to FCz and digitized at 5,000 samples per second with a 500-nV resolution. The single ECG lead included in the EEG cap offers limited visualization of the r-peak of the QRS complex, needed for accurate ballistocardiograph (BCG) correction. Thus, three additional bipolar ECG recordings were taken using a MR-compatible 15-channel amplifier (Brainamp ExG MR, Brain Products GmbH). To reduce BCG artifact by up to 40% (Mullinger et al., 2011), participants were positioned in the MRI scanner so that they were shifted away from the isocenter of the magnetic field by 40 mm, making BCG correction more straightforward.

Data were analog filtered using a 500 Hz band-limiter low-pass filter and a 0.0159 Hz high-pass filter with a 10-sec time constant. Data were recorded with Brain Products Recorder Software, Version 1.x and transferred to the recording computer *via* fiber-optic cable and hardware synchronized to the scanner clock using the Brain Products “Sync Box” (Brain Products GmbH). As recommended in the literature (Mulert and Lemieux, 2010), the MRI sequence parameters were selected to ensure that the gradient artifact would be time stable, and that the lowest harmonic of the gradient artifact (18.52 Hz) would occur at the highest possible frequency and above the spindle band (11–16 Hz). Thus, the MR scan repetition time was set to 2,160 msec, matching a common multiple of the EEG sample time (0.2 msec), the product of the scanner clock precision (0.1 μsec), and the number of slices (40) used.

#### EEG preprocessing

EEG scanner artifacts were removed in several steps. First, an adaptative average template subtraction method (Allen et al., 2000) implemented in Brain Products Analyzer Software, Version 2.x was used and data was downsampled to 250 Hz. Next, r-peaks in the ECG were detected semi-automatically. Each r-peak was visually verified and, when necessary, manually adjusted to correct both false-positive and false-negative r-peak detections. This step is crucial for optimal BCG correction. Next, adaptive template subtraction (Allen et al., 1998) was used to remove BCG artifacts time-locked to the r-peak of the QRS complex. Once the MRI-related artifacts had been corrected, the data was visually inspected and the amplitude of the residual artifacts time-locked to the r-peaks were examined. If the peak of the maximum amplitude of the residual artifact exceeded 3 μV during the QRS complex (e.g., 0–600 msec), an independent component-analysis-based approach (Srivastava et al., 2005; Mantini et al., 2007) was applied to remove any remaining BCG residual artifacts. Lastly, the EEG was re-referenced to the averaged mastoids and a low-pass filter of 60 Hz was applied. Following the artifact correction, sleep stages were scored in accordance with standard criteria (Iber et al., 2007) using the “VisEd Marks” toolbox^1^ for EEGLAB (Delorme and Makeig, 2004).

#### Slow wave detection

Slow waves were automatically detected from Fz, Cz, and Pz during movement artifact-free NREM sleep (N2 and SWS) *via* a period amplitude analysis detection algorithm^2^ similar to that previously described (Bersagliere and Achermann, 2010), adapted for EEGlab (Delorme and Makeig, 2004), and written for MATLAB R2019b (The MathWorks Inc.). First, the EEG signal was band-pass filtered (32nd order Chebyshev Type 2 low-pass filter, 80 dB stopband attenuation, 2.15 Hz frequency cut-off; 64th-order Chebyshev type 2 high-pass filter, 80 dB stopband attenuation, 0.46 Hz frequency cut-off). The cut-off frequencies were selected to achieve minimal attenuation in the band of interest while keeping a good attenuation of the neighboring frequencies. The filters were applied in the forward and reverse directions to achieve zero-phase distortion. Next, half-waves were determined as negative or positive deflections between two consecutive zero crossings in the band-pass filtered signal for frequencies between 0.5 and 2 Hz. Only adjacent half-waves with a peak-to-peak amplitude higher than 75 μV and longer than 0.250 s were considered for the analysis. The latency of the negative peak of each slow wave was extracted.

#### Spindle detection

Sleep spindles were automatically detected from Fz, Cz, and Pz during movement artifact-free NREM sleep (N2 and SWS) using an established (Fogel et al., 2014; Albouy et al., 2015) and validated (Ray et al., 2015) method employing EEGlab-compatible (Delorme and Makeig, 2004) software^3^ written for MATLAB R2019b (The MathWorks Inc.). Detailed processing steps, procedures, and method validation are reported elsewhere (Ray et al., 2015). Briefly, the spindle data were extracted from movement artifact-free, NREM epochs. The detection method (Ray et al., 2015) used a complex demodulation transformation of the EEG signal with a bandwidth of 5 Hz centered about a carrier frequency of 13.5 Hz (i.e., 11–16 Hz) (Iber et al., 2007). The method employs and adaptive amplitude threshold at the 99th percentile on the transformed signal. Following automatic detection, detected spindles were visually verified by a single expert scorer. The variables of interest extracted from this method include spindle amplitude, duration, and density (number of spindles/minute of NREM sleep) for each participant. This method is consistent with several previous EEG-fMRI studies investigating brain activations time-locked to spindles (Laufs et al., 2007; Schabus et al., 2007; Tyvaert et al., 2008; Andrade et al., 2011; Bergmann et al., 2012; Caporro et al., 2012; Fogel et al., 2017a; Fang et al., 2019, 2020; Smith et al., 2020). From the spindle data, the onset of and peak of each spindle event were extracted.

#### Slow wave-spindle coupling

Using the slow wave negative peak latencies and the spindle peak latencies from Fz, Cz, and Pz, we performed coupling detection procedures using the approach originally developed and validated by Mölle et al. (2002, 2011), Clemens et al. (2011), Staresina et al. (2015), Baena et al. (2020), Denis et al. (2021) employing EEGlab-compatible (Delorme and Makeig, 2004) software written for MATLAB R2019b (The MathWorks Inc.). This procedure involved building a time window of 4 s around the negative peaks of the detected slow waves. The spindles were then marked as coupled SW-SP complexes when the spindle peak latency occurred within the 4-s time window of the slow wave peak detected in the same channel as the spindle event. Alternately, spindles were marked as uncoupled when the spindle peak latency occurred outside the 4-s time window. Due to the low number of coupled spindle events on each channel, detections from Fz, Cz, and Pz were merged and used in the final analyses. To address duplicate detection of the same slow wave event by the different channels, we removed events which latency difference was lower than the minimum duration threshold for the half wave (0.125 s). Lag was measured as the distance between the slow wave negative peak and the spindle onset. The lag variance was then calculated for each individual as a measure of coupling strength. Paired sampled *t*-test were used for the comparison of the SW-SP complexes, measured in time bins of 200 ms along the 4 s window.

Additionally, the phase of the bandpass-filtered slow-wave signal in radians at the spindle peak latency was computed. The mean direction of the phase angles for all coupled spindle events were determined using the CircStat toolbox (Berens, 2009). Hilbert transform was applied to extract the preferred phase of SW-SP coupling for each participant averaging all individual events preferred phases. Then, we perform uniformity tests (Rayleigh test) and uniformity using positive slow wave peaks as the predefined mean direction (V-test). From the SW-SP coupling data, and in addition to a list of SW-SP and uncoupled spindle events for each recording, the spindle peak oscillatory frequency and lag for the SW-SP events were extracted.

### MRI acquisition and analysis

#### MRI recording parameters

A 3.0 Tesla Magnetom Prisma MRI system (Siemens) with a 64-channel head coil were used to obtain brain images. Structural T1-weighted MRI images were acquired using a 3-D MPRAGE sequence [repetition time (TR) = 2,300 msec, echo time = 2.98 msec, inversion time = 900 msec, flip angle = 9°, 176 slices, field of view = 256 mm× 256 mm, matrix size = 256 × 256 × 176, voxel size = 1 mm× 1 mm× 1 mm]. During the sleep session, multislice T2*-weighted fMRI images were acquired using an EPI sequence using axial slice orientation (TR = 2,160 msec, echo time = 30 msec, flip angle = 90°, 40 transverse slices, 3-mm slice thickness, 10% interslice gap, field of view = 220 mm× 220 mm, matrix size = 64 × 64 × 40, voxel size = 3.44 mm× 3.44 mm× 3 mm). Up to 2 h of sleep EEG-fMRI data was acquired among all participants during fMRI acquisition.

#### Image preprocessing

Functional images were preprocessed and analyzed using SPM12 (Welcome Department of Imaging Neuroscience)^4^ implemented in MATLAB R2015a (The Mathworks Inc.). fMRI images were corrected for slice acquisition time differences and realigned to correct head motion using rigid body transformation. A mean realigned image was then created from the resulting images. The structural T1 image was coregistered to this mean functional image using a rigid body transformation optimized to maximize the normalized mutual information between the two images. Coregistration parameters were then applied to the realigned BOLD time series. The co-registered structural images were segmented into gray matter, white matter, and cerebrospinal fluid. An average participant-based template was created using DARTEL in SPM12. All functional and anatomical images were spatially normalized using the resulting template, which was generated from the structural scans. Finally, spatial smoothing was applied to all functional images (Gaussian kernel, 8-mm FWHM).

### Statistical analysis

#### First-level (within-subject) GLM

The onset and duration for each SW-SP and uncoupled spindle and slow wave events were identified from the EEG data and were considered events of interest. Friston-24 movement parameters (Friston et al., 1996), the mean white matter intensity, and the mean cerebral spinal fluid intensity for each participant were entered into the model as nuisance variables. A high-pass filter with a cut-off at 128 s was used to remove low frequency drifts from the time series. Brain activations time-locked to each event type (e.g., spindle, SW, and coupled SW-SP events) were estimated using the onset and duration of each event in a fixed-effects GLM using an event-related fMRI design. The BOLD time series data were modeled using the canonical hemodynamic response function (HRF). In addition, to account for the variability in the latency of the peak response and variability in the duration of the peak response, the temporal and dispersion basis function derivatives were also included in the model [this approach is commonly used for similar sleep EEG-fMRI data sets. See (Schabus et al., 2007; Dang-Vu et al., 2008; Urbain et al., 2013) for details]. Consequently, this approach yields an “*informed basis set*,” generating three contrast t-maps, one for the canonical HRF, one for the temporal derivative, and one for the dispersion derivative, for each participant. The resulting contrast images (HRF, temporal, and dispersion derivates) are then entered in a second-level (i.e., group-level), random effects analysis (Penny and Holmes, 2007). Because latency and dispersion derivative effects cannot be computed separately from their canonical HRF at the random effect level (Henson et al., 2002), we computed F tests to jointly test relevant contrasts of parameter estimates. This allowed us to identify cerebral regions in which BOLD response was modulated by the events of interest (e.g., spindles, slow waves, coupled events), using the HRF, and its onset latency and temporal dispersion derivatives (Friston et al., 1998).

#### Second-level (group) GLM

The first-level contrasts maps of coupled SW-SP and uncoupled spindles, and uncoupled SW events comprised of the informed basis set (contrasts for the HRF, temporal, and dispersion basis functions) were entered together into a second-level ANOVA. In this way, we could examine the HRF response for each group comparison without the assumption that the temporal and dispersion functions were constant (Henson et al., 2001; Henson and Penny, 2005). It is necessary to use an ANOVA to include the informed basis set. However, the resulting F-contrast does not provide information about the directionality of the effects. Thus, in order to determine the direction of the effects, the contrast estimates were examined and 95% confidence intervals for the HRF, for each significant cluster. Group-level F-contrasts were generated to examine: (1) brain activation time-locked to SW-coupled spindles, (2) brain activation time-locked to uncoupled spindles; (3) brain activation time-locked to uncoupled slow waves; (4) differences in activation between coupled and uncoupled spindles; (5) differences in activation between coupled and uncoupled slow waves. Finally, a conjunction analysis was also employed to test for similarities between uncoupled spindles and coupled SW-SP and also between uncoupled slow waves and coupled SW-SP. All group-level hypotheses were tested controlling for multiple comparisons using an uncorrected threshold of *p* < 0.001 and then family-wise error (FWE) corrected, at *p* < 0.05, at the cluster-level.

## Results

### Sleep architecture

All participants had more than a total of 14 min of sleep during the recording session in the MRI scanner, and on average, 46.10 min (SD = 23.42 min). The average sleep latency was 8.21 min (SD = 10.64 min), and the average time when participants fell asleep in the scanner was at 22:20 h (SD = 28 min). NREM data from 21 participants were included in the analyses (Table 1). The average duration of NREM sleep was 41.14 min (SD = 19.91 min).

**TABLE 1:** Sleep architecture and spindle parameters for coupled and uncoupled spindles during EEG–fMRI recording.

	*N*	*M*	SD
Sleep architecture			
Wake (min)	19	27.11	21.82
NREM1 (min)	19	5.01	3.82
NREM2 (min)	21	22.72	11.69
SWS (min)	18	16.2	17.51
NREM (min)	21	41.14	19.91
REM (min)	7	14.86	7.97
Total sleep (min)	21	46.1	23.42
Sleep latency (min)	21	8.21	10.64
**Coupled spindles**			
Number	21	271	254
Duration (s)	21	0.71	0.08
Amplitude (μV)	21	34.08	10.02
Density (#/min)	21	4.9	3.36
Frequency (Hz)	21	13.25	0.33
**Uncoupled spindles**			
Number	21	184	127
Duration (s)	21	0.73	0.1
Amplitude (μV)	21	30.5	7.85
Density (#/min)	21	3.67	1.83
Frequency (Hz)	21	13.24	0.42
**Uncoupled slow waves**			
Number	21	926.86	1163.6
Duration (s)	21	0.44	0.05
Amplitude (μV)	21	37.94	5.55
Density (#/min)	21	22.53	28.28
Frequency (Hz)	21	2.74	0.45

NREM, non-rapid eye movement sleep; NREM1, stage 1 sleep; NREM2, stage 2 sleep; SWS, slow wave sleep; REM, rapid eye movement sleep.

### Coupled SW-SP and uncoupled spindle parameters

Coupling status was determined using a previously reported and validated procedure developed by Mölle et al. (2002, 2011), Clemens et al. (2011), Staresina et al. (2015), Baena et al. (2020), Denis et al. (2021). Accordingly, spindles were marked as coupled if the spindle peak latency occurred within a 4 s window built around the slow wave negative peak. EEG trace example of coupled and uncoupled spindles are shown in Supplementary Figure 1. On average, 271 coupled spindles and 184 uncoupled spindles per participant were identified and included in the analyses (Table 1). In line with previous research (Baena et al., 2022; Malerba et al., 2022), spindle amplitude was significantly higher in coupled SW-SP in comparison to uncoupled spindles (*t*_(20)_ = 2.65, *p* = 0.01). There were no differences between coupled SW-SP and uncoupled spindles in terms of the number (*t*_(20)_ = 1.56, *p* = 0.13) duration (*t*_(20)_ = −1.97, *p* = 0.06) or density (*t*_(20)_ = 1.39, *p* = 0.18), hence suggesting they were evenly distributed.

**FIGURE 2:**
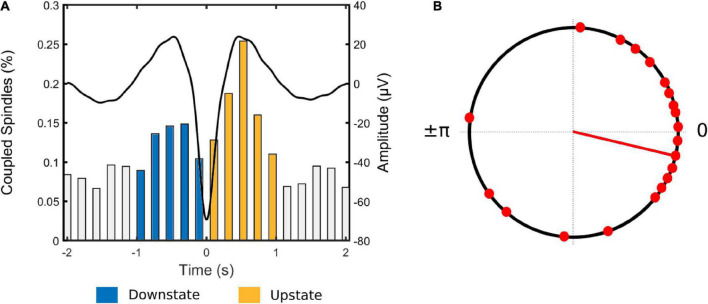
**(A)** Coupled spindle percentage histogram for all participants. Each bar represents the number of coupled spindles detected in an interval of 200 ms divided by the total number of spindles averaged across all participants. Average slow wave coupling window for all participants is superimposed In black. **(B)** Circular plot of preferred phase for each individual (slow wave phase at spindle amplitude maximum). Red dots denote an individual preferred phase (0° slow wave -upstate peak, ± 180° slow wave downstate peak). The direction of the line indicates the preferred direction of the grand average. Most individuals exhibit spindles adjacent to or immediately following the positive slow wave peak at 0°.

Inspection of the temporal distribution of spindles co-occurring within the 4 sec window around the negative peak of the slow wave (Figure 2A) revealed that coupled spindles percentage during the downstate (up-to-down transition interval) ranging from −1 to 0 sec was significantly higher than the preceding interval ranging from −2 to −1 sec (*t*_(20)_ = −5.45, *p* < 0.001). The percentage of coupled spindles during the upstate (down to up interval ranging from 0 to 1 sec), was also higher than the succeeding 1 to 2 sec interval (*t*_(20)_ = 5.89, *p* < 0.001). Finally, the percentage of coupled spindles during the upstate was significantly higher than during the downstate (*t*_(20)_ = −3.19, *p* = 0.005).

Next, to investigate the phase relationship between spindles and slow waves, the slow wave phase at the spindle peak for each SW-SP event was extracted and mean phase angle per individual was computed (Figure 2B). The coupling of spindle events within the slow wave cycle was maximal shortly before the upstate peak in 13 out of 21 participants [0° (slow wave positive peak); *p* < 0.001, V-test]. Further individual-level analyses revealed a non-uniform distribution (*p* < 0.05, Rayleigh test) of the preferred phases of SW-SP in 10 out of 21 participants, suggesting that spindles were coupled to slow waves preferentially adjacent to or immediately following the positive slow wave peak.

### Cerebral activations time-locked to uncoupled (i.e., isolated) spindles

**FIGURE 3:**
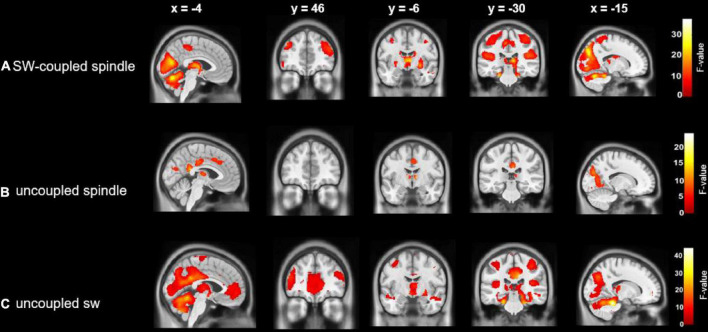
Cerebral activations time-locked to: **(A)** SW-coupled spindles; **(B)** uncoupled spindles and **(C)** uncoupled slow waves. Statistical inferences were performed at *p* < 0.05, FWE corrected at the cluster level.

Activations time-locked to uncoupled spindles were observed in the bilateral thalamus, middle cingulate cortex (MCC), right posterior cingulate cortex (PCC), superior medial frontal cortex (sPFC), and bilateral occipital lobe (Figure 3A and Table 2).

**TABLE 2:** Cerebral activations time-locked to: (A) uncoupled spindles, (B) uncoupled slow waves, and (C) coupled SW-SP.

Hemisphere	Region	Cluster size	Coordinates			Peak-F	Cluster-level (*p*_FWE_)
			*x*	*y*	*z*		
A: Brain activation time-locked to uncoupled spindles							
R	PCC	1,845	4	−40	20	23.77	<0.001
R	MCC		4	−20	32	12.57	
Middle	sPFC		0	28	38	9.13	
Bilateral	Thalamus	231	8	−8	8	13.54	0.044
Bilateral	Occipital lobe	1,592	−20	−76	28	13.46	<0.001
**B:Brain activation time-locked to uncoupled slow waves**							
Bilateral	Parahippocampus	17,601	−22	−30	−20	22.88	<0.001
R	IFG		36	26	0	11.31	
Bilateral	Cerebellum		−18	−38	−26	46.5	
Middle	Vermis		0	−52	−10	24.92	
Bilateral	Cerebellum		−10	−60	−20	24.55	
Bilateral	MCC	14,397	8	−34	34	29.23	<0.001
Bilateral	PCC		−4	−46	30	24.91	
Bilateral	Precuneus		16	−72	36	24.77	
Bilateral	Postcentral gyrus		38	−26	50	15.03	
Bilateral	Occipital lobe		14	−72	40	24.18	
L	MFG	6,287	−46	20	38	14.86	<0.001
L	Temporal pole		−52	6	−10	14.14	
L	IFG		−44	46	12	13.71	
Bilateral	ACC	2,487	12	40	−2	13.6	<0.001
Bilateral	STG	674	42	−34	16	11.29	0.001
**C: Brain activation time-locked to coupled SW-SP spindles**							
Bilateral	Occipital lobe	17,684	−18	−76	32	30.71	<0.001
Bilateral	Postcentral gyrus		40	−36	54	17.33	
Bilateral	Putamen	764	28	−10	−4	20.01	<0.001
R	STG	1,296	50	−26	22	14.79	<0.001
Bilateral	MFG	2,679	−52	32	20	13.65	<0.001
Bilateral	IFG		−34	28	−2	12.52	<0.001
L	Parahippocampus	10,681	−24	−28	−24	10.7	<0.001
R	hippocampus		12	−34	8	18.68	
Bilateral	Cerebellum		−22	−34	−26	36.56	
L	Vermis		−2	−70	−18	29.24	
Bilateral	Cerebellum		−26	−38	−30	25.4	
R	Thalamus		6	−8	6	22.73	

MCC, middle cingulate cortex; PCC, posterior cingulate cortex; ACC, anterior cingulate cortex; IFG, inferior frontal gyrus; MFG, middle frontal gyrus; STG, superior temporal gyrus. sPFC, superior prefrontal cortex; L/R, Left/Right. Statistical inferences were performed at a threshold of *p* < 0.001 uncorrected at the whole-brain level and *p* < 0.05, FWE corrected at the cluster level.

### Cerebral activations time-locked to uncoupled (i.e., isolated) slow waves

Uncoupled slow waves showed a very similar pattern of activation (Figure 3C and Table 2) as compared to coupled SW-SP (see below, and Figure 3B), including the occipital lobe, bilateral postcentral gyrus, bilateral superior temporal gyrus (STG), bilateral IFG, bilateral thalamus, anterior cingulate cortex, PCC and bilateral hippocampus (Figure 3C and Table 2).

### Cerebral activations time-locked to coupled SW-SP

Activations time-locked to SW-SP were observed in the occipital lobe, bilateral postcentral gyrus, bilateral STG, bilateral middle frontal gyrus (MFG), bilateral inferior frontal gyrus (IFG), bilateral thalamus, bilateral putamen, and hippocampus (Figure 3B and Table 2).

### Similarities and differences between SW-SP and uncoupled (i.e., isolated) spindles

**FIGURE 4:**
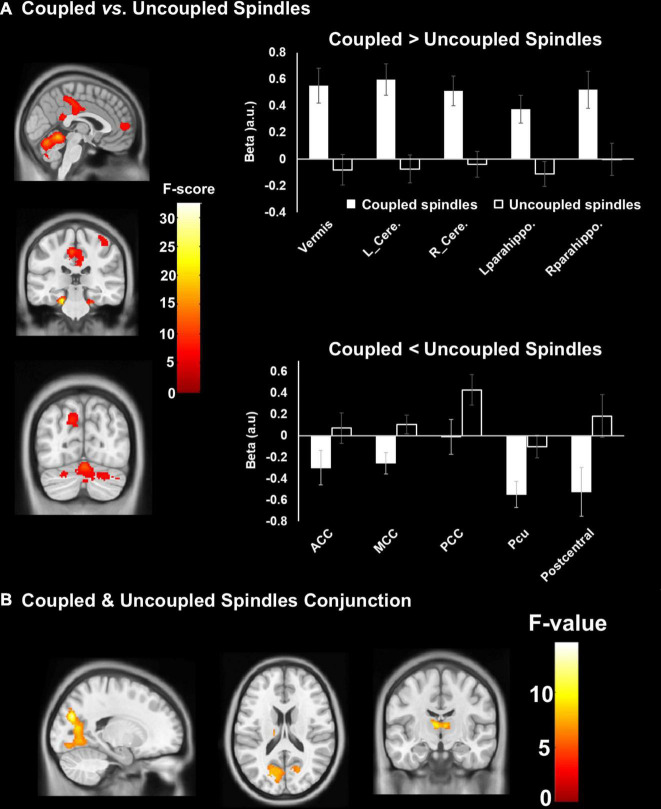
Differences in cerebral activation between **(A)** coupled vs. uncoupled spindles, and, **(B)** conjunction between coupled and uncoupled spindles. Statistical inferences were performed at *p* < 0.05, FWE was corrected at the cluster level.

A conjunction analysis showed that both SW-coupled and uncoupled spindles presented robust activation of the bilateral thalamus and bilateral occipital lobe (Figure 4B). Further comparisons showed differences in fMRI activity between coupled SW-SP and uncoupled spindles were observed in the cerebellum, bilateral parahippocampus, anterior/middle/posterior cingulate cortex (ACC/MCC/PCC), precuneus and right postcentral gyrus (Figure 4A and Table 3). Inspection of the HRF contrasts verified that coupled SW-SP were associated with larger changes in fMRI signal than uncoupled spindles in the cerebellum and bilateral parahippocampus (Figure 4A). Uncoupled spindles on the other hand, showed greater activation in the cingulate cortex, precuneus (Pcu) and right postcentral gyrus compared to coupled SW-SP (Figure 4A). Thus suggesting that SW-SP coupled events differ from uncoupled spindles, notably in hippocampal regions, but share recruitment of areas critical for generating these events, and in sleep maintenance, such as the thalamus. BOLD-signal peristimulus plots for selected areas are included in Supplementary Figure 2.

**TABLE 3:** Differences in cerebral activations between: (A) coupled SW-SP vs. uncoupled spindles, and (B) coupled SW-SP and uncoupled slow waves.

Hemisphere	Region	Cluster-size	Coordinates			Peak-F	Cluster-level (*p*_FWE_)
			*x*	*y*	*z*		
A: Brain activation differences: Coupled SW-SP *vs*. uncoupled spindles							
Bilateral	MCC	1,410	−6	−28	46	13.32	<0.001
R	PCC		6	−44	20	11.96	
L	ACC	332	−8	60	6	9.47	0.011
R	Postcentral gyrus	437	42	−28	66	8.47	0.003
L	Precuneus	290	−14	−74	36	10.36	0.019
Bilateral	Parahippocampus	5,134	−22	−28	−22	16.61	<0.001
Middle	Vermis		0	−52	−10	18.93	
R	Cerebellum		26	−34	−28	17.43	
L	Cerebellum		−22	−64	−28	17.07	
**B: Brain activation differences: Coupled SW-SP *vs*. uncoupled slow waves**							
Bilateral	Precuneus	572	−14	−46	32	10.54	0.002
Bilateral	PCC		−2	−46	32	10.17	

MCC, middle cingulate cortex; PCC, posterior cingulate cortex; ACC, anterior cingulate cortex; IFG, inferior frontal gyrus; MFG, middle frontal gyrus; STG, superior temporal gyrus. L/R, Left/Right. Statistical inferences were performed at a threshold of *p* < 0.001 uncorrected at the whole-brain level and *p* < 0.05, FWE corrected at the cluster level.

### Similarities and differences between SW-SP and uncoupled (i.e., isolated) slow waves

**FIGURE 5:**
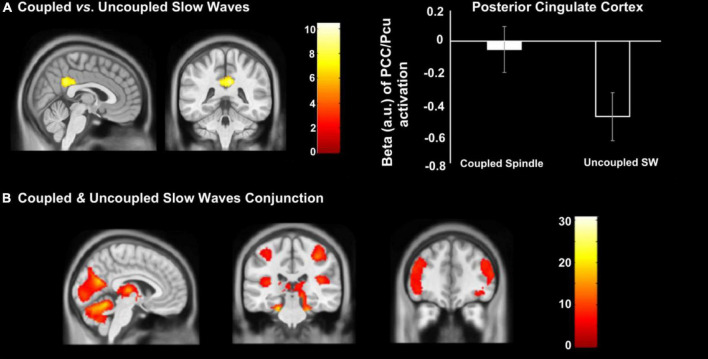
Differences in cerebral activation between **(A)** coupled vs. uncoupled slow waves, and **(B)** conjunction between coupled and uncoupled slow waves. Statistical inferences were performed at *p* < 0.05, FWE was corrected at the cluster level.

Conjunction analysis revealed the common areas in both coupled SW-SP and uncoupled slow waves, including the occipital lobe, bilateral postcentral gyrus, bilateral superior temporal gyrus (STG), bilateral IFG, bilateral thalamus and bilateral hippocampus (Figure 5B). Only the PCC was significantly greater in coupled SW-SP than that in response to uncoupled SW (Figure 5A and Table 3). Thus, suggesting that SW-SP coupled events are mostly indistinguishable from uncoupled slow waves, except for the PCC.

## Discussion

In the present study, we investigated functional dissociations in cerebral activation that are specifically related to the occurrence of SW-SP coupling. Previously, converging evidence from multiple studies has implicated SW-SP coupling as an important mechanism supporting the consolidation of new information into long term memory (Novitskaya et al., 2016; Latchoumane et al., 2017; Bergmann and Born, 2018; Baena et al., 2020; Ngo et al., 2020). However, no study to date has specifically examined the patterns of functional brain activations of coupled SW-SP. The results of our study revealed a clear differentiation between regions recruited during coupled SW-SP versus uncoupled spindles as well as during, coupled SW-SP vs. uncoupled SWs, namely: (1) coupled SW-SP uniquely activated bilateral middle frontal gyrus and the bilateral putamen, (2) uncoupled spindles uniquely activated the MCC and sPFC, and, (3) uncoupled slow waves uniquely activated the ACC. Areas that were activated in all three contexts included the bilateral thalamus and bilateral occipital lobe. Furthermore, compared to uncoupled spindles, coupled SW-SP showed relatively greater activation of the cerebellum and bilateral parahippocampal cortex, and relatively lower activation of the ACC, MCC, PCC, precuneus, and right postcentral gyrus. Activation of the PCC was observed in both uncoupled spindles as well as uncoupled slow waves, while coupled slow waves showed greater deactivation in the PCC when compared to uncoupled slow waves. Activation observed in both SW-SP and uncoupled slow waves included bilateral postcentral gyrus, STG, IFG, and interestingly, the bilateral hippocampus. Taken together, these results support the view that the co-occurrence of spindles and slow waves acts as a unique neurophysiological mechanism, resulting in the activation of brain areas not observed when either spindles or slow waves occur in isolation.

Our results are consistent with previous studies using EEG-fMRI to measure cerebral activation and functional connectivity associated with sleep spindles (Schabus et al., 2007; Andrade et al., 2011; Caporro et al., 2012; Fogel et al., 2017b). The consensus from these studies suggests that the recruitment of the hippocampus associated with sleep spindles is a spindle-driven phenomenon. Our current results advance our understanding of this process and suggest that this relationship is more nuanced in important ways; in the absence of slow waves, spindles do not show hippocampal activation, and yet slow waves show hippocampal activation in the absence of spindles. Moreover, there were no specific brain structures that showed activation common to both coupled SW-SP and uncoupled spindles, except for those that were also activated during uncoupled slow waves and are involved in the generation of these events, and in sleep maintenance (i.e., thalamus). However, both coupled SW-SP and uncoupled slow waves showed hippocampal activation, whereas uncoupled spindles did not. In light of these findings, we postulate that hippocampal activation (and possibly, *reactivation* of brain areas) during sleep is not unique to spindles, but primarily driven by slow waves, and that the co-occurrence of spindles allows the recruitment of frontal areas, creating a unique functional activation pattern, which may be critical for the hippocampal-neocortical dialogue that preferentially occurs during sleep, and is known to support memory consolidation processes (Rasch and Born, 2013). However, the specific behavioral outcomes for reactivation of SW-SP coupling for memory consolidation, *per se*, remain to be directly investigated.

According to our results, the bilateral putamen were uniquely activated during coupled SW-SP. Putamen has been shown to play a central role in the consolidation of motor skills during sleep (Fogel et al., 2014; King et al., 2017; Vahdat et al., 2017) and also to be correlated with high-level cognitive functions beyond organization and execution of movements (Ketteler et al., 2008; Mestres-Missé et al., 2010). Based on related studies we speculate that activation of the putamen in this context may serve as a marker for coupling-related memory consolidation during sleep, perhaps *via* memory trace reactivation (Fogel et al., 2017b). Interestingly, while hippocampal activation did not differ between coupled and uncoupled spindles, coupled spindles did show greater activation of the parahippocampus and cerebellum. Thus, it may be that sleep spindles *per se* do not support hippocampal functions in isolation. Rather, this may preferentially occur when spindles and slow waves are coupled. This is consistent with the extant literature which suggests that SW-SP coupling supports the transfer of slow-wave-driven hippocampal signals into cortical and cerebellar structures, ultimately integrating memories into existing schema encoded in these areas. For example, slow wave-driven reactivation of a freshly encoded memory trace in the hippocampus may serve to maintain the integrity of the new memories’ neural code (Alger et al., 2012; Schlichting and Preston, 2015; Moyano et al., 2019). Coupling of spindles with slow waves then provides an opportunity for integration of the memory into the parahippocampal cortex to be output to other brain areas for long-term storage, where contextual information is processed and eventually integrated into a predominantly cortical, and more schematized memory trace (Schlichting and Preston, 2015; Kitamura et al., 2017; Noack et al., 2021). Moreover, the parahippocampal gyrus is of specific importance for memory of spatial configurations of objects, but not necessarily their identity (Bohbot et al., 2015); thus, suggesting that spindles may be more directly involved in memory processing for spatial aspects of memory processing (Albouy et al., 2013). While we cannot conclude specific mnemonic functions from the current study, this study lays the groundwork for future studies to directly investigate this at the behavioral level.

Uncoupled slow wave events showed an activation pattern very similar to the coupled SW-SP events with exception of the PCC region. This region has been previously associated with memory consolidation processes linking episodic and semantic information (Maguire et al., 1999). More recent studies using MRI confirm this integrating role, further detailing that PCC plays a crucial role in memory schema formation (Bird et al., 2015). Our results thus suggest that although hippocampal activation is shared between coupled SW-SP and uncoupled SWs, the lower level of PCC deactivation during coupled SW-SP could indicate that memory integration and schema formation may only be attributable to coupled SW-SP events. This, however, remains to be directly investigated.

There are several limitations worth mentioning in the present study. It was not possible to subdivide spindles into slow spindles (11–13 Hz) and fast spindles (13–16 Hz), nor was it possible to subdivide spindles from NREM2 and SWS due to the limited sleep duration and number of events recorded while participants were sleeping in the MRI scanner. In addition, we did not directly investigate any relationship between spindles, slow waves and SW-coupled spindles following new learning or associated with cognitive abilities. This remains to be directly investigated in future studies specifically designed to assess the reactivation of memory traces and the related behavioral outcomes. However, this study does represent a necessary first step in this line of investigation.

In summary, we investigated differences in cerebral activation linked to SW-coupled spindles and both their differences and similarities with uncoupled spindles and uncoupled SWs. The results demonstrate a clear differentiation in brain areas recruited time-locked to SW-coupled spindles and uncoupled spindles supporting that both events are functionally dissociable. By contrast, these results show largely similar activation patterns between SW-coupled spindles and uncoupled slow waves. For the first time, we provide direct evidence of hippocampal activation linked specifically to coupled SW-SP complexes in comparison to isolated spindles. This finding is consistent with other studies that show coordinated activity of these precisely timed complexes is specifically involved in memory consolidation *via* reactivation. Furthermore, and surprisingly, the present study revealed that this pattern of activation may be driven more so by slow waves than spindles. We propose that it is possible that hippocampal reactivation during sleep is not uniquely related to spindles. Rather, this pattern of activation may be primarily driven by slow waves. Our results also suggest that SW-spindle coupling may be critical for functional recruitment of the putamen. Importantly, the co-occurrence of spindles and slow waves may allow the recruitment of frontal areas, which may be critical for the hippocampal-neocortical dialogue that preferentially occurs during sleep. Future studies linking coupling-driven activation with memory performance are needed to further elucidate the dissociable contribution of isolated spindles, isolated slow waves and SW-SP complexes to memory consolidation and other functions.

## Author’s note

Sleep is known to aid in memory consolidation. This involves transfer of information from the hippocampus to the neocortex and is facilitated by slow wave – spindle – hippocampal ripples complexes during sleep. However, no study to date has specifically examined if the patterns of functional brain activations of isolated spindles differ from slow wave coupled spindles. Here, we found a clear differentiation in brain areas recruited for each event. Coupled spindles recruited frontal areas and parahippocampus in comparison to uncoupled spindles. This suggests that coupled spindles may be critical for the hippocampal-neocortical dialogue that occurs during sleep. Our results lay the groundwork for future studies to differentiate between spindles according to coupling status.

## Data availability statement

The datasets presented in this article are available by request to the corresponding author, following required ethical approval and inter-institutional data sharing agreements. Requests to access the datasets should be directed to SF, sfogel@uottawa.ca.

## Ethics statement

The studies involving human participants were reviewed and approved by Western University Health Science Research Ethics Board. The patients/participants provided their written informed consent to participate in this study.

## Author contributions

SF and AO designed the study and supervised the research. SF, ZF, DB, and LR carried out the research and collected the data and contributed to the data analyses. DB, SF, and LR prepared the figures and wrote the manuscript. AG, DS, JD, and SF revised the manuscript. All authors discussed the results and commented on the manuscript.

## Footnotes

1
https://github.com/jadesjardins/vised_marks

2
https://github.com/stuartfogel/Period-Amplitude-Analysis

3
https://github.com/stuartfogel/detect_spindles

4
www.fil.ion.ucl.ac.uk/spm/software/spm12/
