# Supplementary figures and images for: Functional differences in cerebral activation between slow wave-coupled and uncoupled sleep spindles
Source: Front Neurosci. 2023 Jan 18;16:1090045. doi: 10.3389/fnins.2022.1090045 (PMC9889560; doi:10.3389/fnins.2022.1090045)

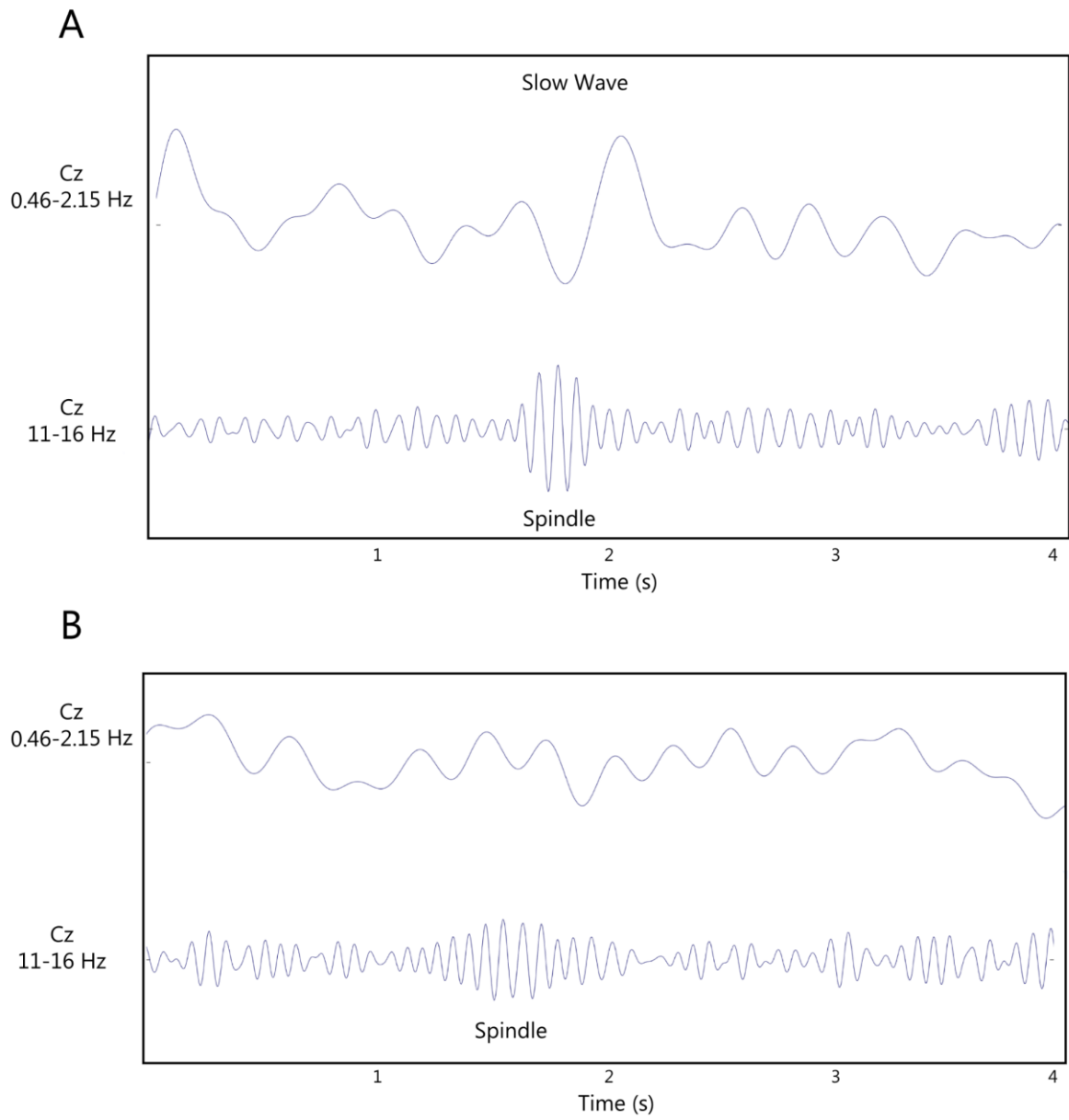

**Figure S1.** EEG trace example for **(A)** coupled SW-spindle and **(B)** uncoupled spindle events.

Supplement: Supplementary file 1 [file Image_1.PDF]
