# Supplementary material for: Functional differences in cerebral activation between slow wave-coupled and uncoupled sleep spindles
Source: Front Neurosci. 2023 Jan 18;16:1090045. doi: 10.3389/fnins.2022.1090045 (PMC9889560; doi:10.3389/fnins.2022.1090045)
Supplement: Supplementary file 2 [file Image_2.PDF]

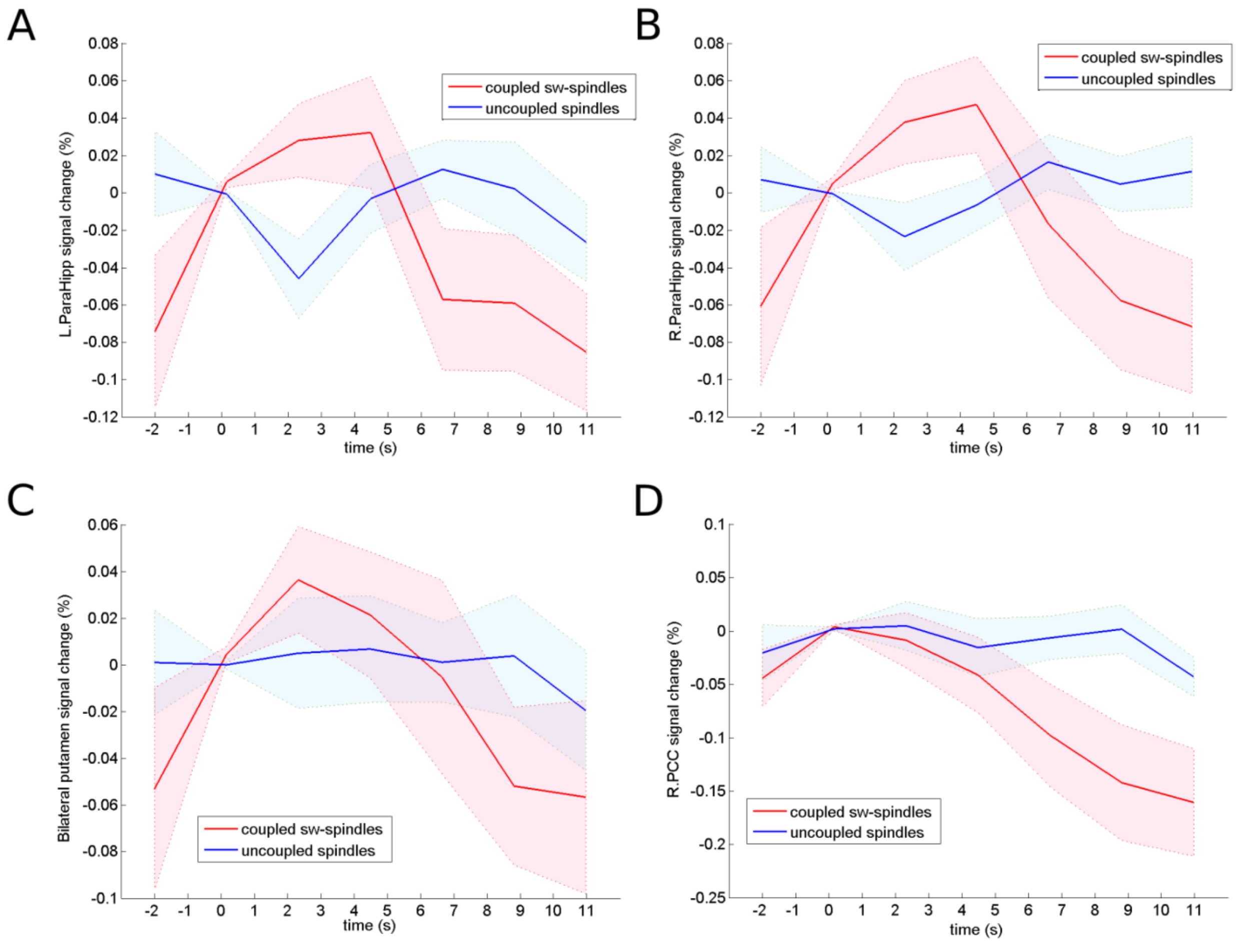

Figure S2. BOLD-signal peristimulus plots in response of coupled SW-spindles (red) and uncoupled spindles (blue) for: **(A)** left parahippocampus; **(B)** right parahippocampus; **(C)** bilateral putamen and **(D)** right posterior cingulate cortex.
